# miR-143 Regulation of Prostaglandin-Endoperoxidase Synthase 2 in the Amnion: Implications for Human Parturition at Term

**DOI:** 10.1371/journal.pone.0024131

**Published:** 2011-09-07

**Authors:** Sun Young Kim, Roberto Romero, Adi L. Tarca, Gaurav Bhatti, JoonHo Lee, Tinnakorn Chaiworapongsa, Sonia S. Hassan, Chong Jai Kim

**Affiliations:** 1 Perinatology Research Branch, National Institute of Child Health and Human Development/National Institutes of Health/Department of Health and Human Services, Bethesda, Maryland, United States of America; 2 Perinatology Research Branch, National Institute of Child Health and Human Development/National Institutes of Health/Department of Health and Human Services, Detroit, Michigan, United States of America; 3 Department of Obstetrics and Gynecology, Wayne State University School of Medicine, Detroit, Michigan, United States of America; 4 Department of Computer Science, Wayne State University, Detroit, Michigan, United States of America; 5 Department of Pathology, Wayne State University School of Medicine, Detroit, Michigan, United States of America; Universidade de Sao Paulo, Brazil

## Abstract

**Background:**

The human amnion plays a pivotal role in parturition. Two of its compartments, the placental amnion and the reflected amnion, have distinct transcriptome and are functionally coordinated for parturition. This study was conducted to determine the microRNA (miRNA) expression pattern and its significance in the placental amnion and the reflected amnion in association with labor at term.

**Methodology/Principal Findings:**

MicroRNA microarray, real-time quantitative RT-PCR (qRT-PCR), and miRNA in situ hybridization analyses of the placental amnion and the reflected amnion (n = 20) obtained at term were conducted. Luciferase assay, transfection, and qRT-PCR analyses of primary amnion epithelial cells (AECs) and amnion mesenchymal cells (AMCs) were performed. MicroRNA microarray analysis demonstrated differential expression of 32 miRNAs between the placental amnion and the reflected amnion after labor. Thirty-one (97%) miRNAs, which included miR-143 and miR-145, a cardiovascular-specific miRNA cluster, were down-regulated in the reflected amnion. Analyses of miR-143 and miR-145 by qRT-PCR confirmed microarray results, and further demonstrated their decreased expression in the reflected amnion with labor. Interestingly, expression of miR-143 and miR-145 was higher in AMCs than in AECs (*p*<0.05). Luciferase assay and transfection confirmed miR-143 binding to 3′ UTR of prostaglandin-endoperoxidase synthase 2 (PTGS2) mRNA and miR-143 regulation of PTGS2 in AMCs.

**Conclusions:**

We report region-specific amniotic microRNAome and miR-143 regulation of PTGS2 in the context of human labor at term for the first time. The findings indicate that miRNA-mediated post-transcriptional regulation of gene expression machinery in the amnion plays an important role in the compartments (placental amnion vs reflected amnion) and in a cell type-specific manner (AECs vs AMCs) for parturition.

## Introduction

Human parturition has distinct features unique from other species, and biological activation of the chorioamniotic membranes is a key component of a cascade of events leading to labor at term [1], [2]. The amnion is the inner layer of the chorioamniotic membranes which play critical roles in the maintenance of pregnancy and the initiation of parturition [3]–[5]. It is composed of a monolayer of amnion epithelial cells (AECs) and amnion mesenchymal cells (AMCs) [6] which have phenotypic characteristics of myofibroblasts or macrophages [7]–[9]. Many studies have elegantly addressed both mechanical and biochemical properties of the amnion [10]–[13]. Although it is a single structure, the amnion is anatomically divided into three distinct compartments: placental amnion (amnion over the placental disc), reflected amnion (amnion of the free chorioamniotic membranes), and umbilical amnion (amnion of the umbilical cord) [14]. We have reported that there is an intriguing, stark difference between the placental amnion and the reflected amnion in the transcriptome [15]. Furthermore, the analyses of expression in amniotic prostaglandin-endoperoxidase synthase 2 (PTGS2) and prostaglandin E_2_ (PGE_2_) indicated spatial and functional coordination between the placental amnion and the reflected amnion for parturition at term [16]. The placental amnion is responsible for increasing tonic production of prostaglandins with progression of gestation, whereas there is a surge of PTGS2 synthesis and PGE_2_ production in the reflected amnion with labor at term.

MicroRNAs (miRNAs) are a class of non-coding small RNAs ranging from 18 to 25 nucleotides in size, involved in post-transcriptional regulation of gene expression by mRNA degradation or translational repression through binding to the complementary sequence of the 3′-untranslated region (3′ UTR) of their target mRNA [17], [18]. As more than 1,000 miRNAs are predicted in the human genome [19], which would target approximately 60% of mammalian genes [20], the role of miRNAs is profound in various physiologic and pathologic processes such as development and cancer [21]–[23]. Not surprisingly, recent studies have reported significant changes in placental miRNA expression associated with placental development and pregnancy complications such as preeclampsia [24], [25], fetal growth restriction [26], and acute chorioamnionitis [27]. Changes in miRNA expression patterns in the chorioamniotic membranes also clearly indicated an important role for miRNA in human parturition; yet, amnion-specific characterization of a role for miRNAs has not been investigated. Considering the crucial nature of the amnion in parturition, elucidation of post-transcriptional regulation by miRNAs in the amnion would provide important information about the biology of parturition.

The purpose of this study was to compare miRNA expression patterns in the human placental amnion and the reflected amnion associated with labor at term and thereby to elucidate post-transcriptional gene expression regulatory mechanisms involved in human parturition.

## Materials and Methods

### Patients and tissue collection

Tissue samples of placental amnion and reflected amnion were collected by blunt dissection as described previously [15]. Paired samples for miRNA microarray analysis were obtained from women at term not in labor (TNL, n = 5) and at term in labor (TIL, n = 5). To confirm microarray results by real-time quantitative reverse transcription PCR, an additional 11 pairs of placental amnion and reflected amnion from TNL (n = 5) and TIL (n = 6) cases were used. The indication for Cesarean section in 8 out of 10 term not in labor cases was previous Cesarean delivery. The other two cases included a case of fetal malpresentation (frank breech presentation) and a case of suspected fetal macrosomia. The patient demographics and clinical information are summarized in Table 1. For primary AEC and AMC cultures, reflected amnion tissues were obtained from term placentas delivered by caesarean section in the absence of labor. All patients delivered at Hutzel Women's Hospital, Detroit, Michigan, and provided written informed consent. The collection and use of materials for research purposes were approved by the Institutional Review Boards of Wayne State University and the *Eunice Kennedy Shriver* National Institute of Child Health and Human Development, National Institutes of Health, U.S. Department of Health and Human Services.

**Table 1:** Patient demographics and clinical information of cases used for microarray and confirmation analyses.

	Term in labor	Term no labor	
	n = 11**	n = 10	
Maternal age (years)*	22 (20–39)	29 (19–38)	NS
Race (%)			NS
Black	81.8	60.0	
White	0.0	30.0	
Hispanic	9.1	0.0	
Others	9.1	10.0	
Parity (Nullipara, %)	0.0	20.0	NS
Chronic hypertension (%)	0.0	10.0	NS
Diabetes mellitus (%)	0.0	0.0	NS
Delivery mode (C/S)	0.0	100.0	<0.001
Gestational age at delivery (weeks)*	39.4 (37.1–40.7)	38.9 (37.4–39.4)	NS
Birth Weight (g)*	3270 (2915–3945)	3532.5 (2545–4655)	NS
Placental Weight (g)*	500 (383–660)	527.5 (360–639)	NS
Labor duration (hr)*	8.5 (1.0–21.0)	0.0 (0.0–0.0)	<0.001
Anesthesia before delivery (%)	72.7	100	NS
Oxytocin use before delivery (%)	27.3	0	NS

*, median (range).

**, The number of term in labor cases is 11 because 6 additional cases were used for qRT-PCR analysis.

NS, not significant.

### MicroRNA microarray

Amnion tissues were liquid nitrogen-pulverized using a mortar and pestle, and total RNA was isolated using Trizol (Invitrogen, Carlsbad, CA). The quality of the total RNA was verified using the Agilent 2100 Bioanalyzer (Agilent Technologies, Wilmington, DE). All samples were DNased and cleanup-purified with an RNeasy minicolumn (Qiagen, Valencia, CA). Total RNA (300 ng) from each sample and reference (pooled RNA) were labeled with Hy3™ and Hy5™, respectively, using the miRCURY™ LNA Array Power Labeling Kit (Exiqon, Vedbaek, Denmark) according to the manufacturer's instructions. The Hy3™-labeled sample and a Hy5™-labeled reference RNA sample were mixed pair-wise and hybridized onto the miRCURY™ LNA array version 11.0 (Exiqon). Twenty RNA samples were analyzed individually. The array platform contains capture probes targeting all human, mouse, and rat miRNAs registered in the miRBASE version 13.0 at the Wellcome Trust Sanger Institute, Hinxton, Cambridge, United Kingdom. The hybridization was performed according to the miRCURY™ LNA array manual using a Tecan HS4800™ Pro hybridization station (Tecan Austria GmbH, Grödig, Austria). All microarray data is MIAME compliant and that the raw data has been deposited in a MIAME compliant database (GEO; accession number GSE27441) as detailed on the MGED Society website http://www.mged.org/Workgroups/MIAME/miame.html.

### Real-time quantitative reverse transcription PCR (qRT-PCR)

Total RNA was reverse transcribed using the TaqMan MicroRNA Reverse Transcription Kit (Applied Biosystems, Foster City, CA) and the Improm-II Reverse Transcription System (Promega, Madison, WI) for miRNA and mRNA analyses, respectively. All PCR analyses were carried out by TaqMan assays (Applied Biosystems). For miRNA expression analysis, miR-143 (002146) and miR-145 (002149) TaqMan assays were used with 5S ribosomal RNA (4332078) as a normalizer. For the analysis of PTGS2 mRNA expression (Hs 01573477_g1), RPLPO (large ribosomal protein) was used for normalization. PCR reactions were done using the 7500 Fast Real-Time PCR System (Applied Biosystems).

### MicroRNA in situ hybridization

For in situ hybridization, a 5′-DIG labeled mercury miR-143 LNA probe (Exiqon, Cat. 38515-01) was 3′-end double labeled using a DIG Oligonucleotide Tailing Kit (Roche, Mannheim, Germany). A scrambled LNA detection probe was used as a negative control (Exiqon, Cat. 99004-01). Ten-µm-thick frozen tissue sections were obtained on silanized slides and fixed with 4% (wt/vol) paraformaldehyde for 10 min. After fixation, sections were acetylated for 10 min using the acetylation solution (1.34% of triethanolamine, 0.2% of HCl, 0.6% of acetic anhydride). Acetic anhydride was added to the solution immediately before use. PBS was used for washing after each step. Following the proteinase K (5 µg/ml) treatment at room temperature for 5 min, sections were incubated with a hybridization buffer containing the probe (2 ρmol/slide) for 5 min at 60°C; then it was hybridized for 15 h at 37°C. Probes were denatured at 65°C for 5 min and then quickly chilled on ice before application. A hybridization buffer was composed of 50% formamide, 5× SSC, 5× Denhardt's solution, 200 µg/ml yeast RNA, 500 µg/ml salmon sperm DNA, 2% blocking reagents (Roche), 0.25% CHAPS, and 0.5% Tween 20. After hybridization, slides were washed with 0.2× SSC and 2% BSA at 4°C for 5 min, and incubated with an anti-DIG-alkaline phosphatase antibody (1∶500; Roche) at 37°C for 30 min. The signal was detected using a fast red substrate system (DAKO, Carpinteria, CA), and counterstaining and mounting were carried out using Prolong Gold Antifade Reagent with DAPI (Invitrogen).

### Primary amnion cell culture

The reflected amnion was cut into 2 cm×2 cm pieces. To isolate AMCs, approximately one-third of the obtained amnion fragments were transferred into two tubes containing 25 ml of collagenase A (1 mg/ml) and incubated at 37°C with gentle shaking for 3 h. The digests were then filtered through a 100-micron nylon mesh, and centrifuged at 200×g for 10 min. AMCs were suspended in DMEM (Mediatech, Herndon, VA) containing 10% fetal bovine serum and antibiotics. The remaining two-thirds of the amnion fragments were then placed in 10 ml of 0.05% (w/v) trypsin/EDTA (Invitrogen) and gently shaken for 30 sec to isolate AECs; the fragments were transferred to two new tubes and 15 ml of trypsin/EDTA were added, followed by incubation at 37°C with gentle shaking for 10 min. This trypsin digestion supernatant was discarded; amnion fragments were transferred into a new tube containing 25 ml of fresh trypsin/EDTA solution and were incubated for an additional 40 min at 37°C. After digestion, the supernatant was mixed with an equal volume of DMEM and centrifuged for 10 min at 200×g. The pellet was resuspended in DMEM. Remaining amnion trypsin digests were additionally treated with 25 ml of trypsin/EDTA at 37°C for 40 min. Collected AECs were pooled with the previous cell suspension [28]. AECs and AMCs were kept in DMEM, and all experiments were performed with the cells at passage 2 and passage 4.

### Transfection

For the transfection with miR-143 mimic and inhibitor, 5×10^5^ of AMCs and 1×10^6^ of AECs were split in 6-well plates, kept overnight, and transfected with miRIDIAN miR-143 mimic (50 nM; Dharmacon, Lafayette, CO; C-301057-01-0005) or miR-143 hairpin inhibitor (200 nM; IH-301057-02-0005) using Lipofectamine 2000 (Invitrogen). The miRIDIAN microRNA negative controls (mimic; CN-001000-01-05, hairpin inhibitor; IN-001005-01-05) were used at equimolar concentrations. To estimate transfection efficiency prior to the transfections using miR-143 mimic and inhibitor, the cells were transfected with Cy3-labeled Pre-miR™ negative control #1 (Applied Biosystems) using Lipofectamine 2000, and visualized under an immunofluorescence microscope.

### Generation of PTGS2 3′ UTR reporter construct

The PTGS2 3′ UTR carrying a putative miR-143 binding site was PCR amplified, sequence confirmed, and cloned into a SpeI and HindIII site of the pMIR-REPORT™ miRNA Expression Reporter Vector (Ambion, Austin, TX). The PCR was performed using an upstream primer (5′- CAAGATGGATGCAAAGAGGCTAGTGCCTCA-3′) bearing a SpeI site and a downstream primer (5′-AGAGGTAACCCCAAAGAAGATATACTGATT-3′) bearing a HindIII site. The PCR product of 355 bp was purified from 1% agarose gel after electrophoresis with a PureLink™ Quick Gel Extraction Kit (Invitrogen), and its sequence was verified by DNA sequencing using ABI 3100 sequencer (Applied Biosystems).

### Luciferase assay

To assess miR-143 binding to PTGS2 mRNA 3′ UTR in AECs, 1×10^6^ cells were transfected with 200 ng of pMIR-REPORT plasmid or 200 ng of pMIR-REPORT-PTGS2 3′ UTR, 10 ng of Renilla luciferase reporter pSV40-RL (transfection control; Promega), and miRIDIAN miR-143 mimic (100 nM) or miR-143 hairpin inhibitor (50 nM) or equal amounts of negative controls using Lipofectamine 2000 (Invitrogen). At 48 h after transfection, luciferase assays were carried out using the Dual-Luciferase Reporter Assay System (Promega). For luciferase analysis of AMCs, 5×10^5^ cells were transfected with 500 ng of pMIR-REPORT plasmid or 500 ng of pMIR-REPORT-PTGS2 3′ UTR , 10 ng of Renilla luciferase reporter pSV40-RL, and miRIDIAN miR-143 mimic (100 nM) or miR-143 hairpin inhibitor (50 nM) or equal amounts of negative controls using Lipofectamine 2000 (Invitrogen). At 48 h after transfection, luciferase assays were carried out using the Dual-Luciferase Reporter Assay System (Promega) according to the manufacturer's instructions.

### Immunofluorescence staining

The AECs and AMCs were grown in DMEM on coverslips placed in 6-well plates. Cells were then fixed with 4% of paraformaldehyde and permeabilized with 0.25% triton X-100, and incubated with 5% (w/v) BSA in PBS for 30 min at room temperature. Cells were immunostained using a rabbit polyclonal anti-cytokeratin-7 (1∶25; Abcam, Cambridge, MA) or a mouse monoclonal anti-type I procollagen (1∶25; Developmental Studies Hybridoma Bank at the University of Iowa City, IA) or mouse monoclonal anti-smooth muscle actin alpha (Vector, Burlingame, CA). Alexa Fluor® 594 goat anti-rabbit IgG and Alexa Fluor® 488 goat anti-mouse IgG (Invitrogen) were secondary antibodies, respectively. Stained cells were mounted with Prolong Gold Antifade Reagent with DAPI (Invitrogen), and were examined using a Leica TCS SP5 spectral confocal system (Leica Microsystems Inc., Wetzlar, Germany).

### Immunoblotting

Total proteins were isolated using RIPA buffer (Sigma-Aldrich) containing a protease inhibitor cocktail (Roche). Fifteen µg of protein were subjected to 10% SDS-polyacrylamide gel electrophoresis, and electro-transferred onto nitrocellulose membranes. Membranes were blocked with 5% nonfat dry milk in Tris-buffered saline containing 0.1% v/v Tween 20, and were probed overnight at 4°C with a rabbit polyclonal anti-PTGS2 (1∶500; Cell Signaling, Danvers, MA) and a mouse monoclonal anti-β-actin (1∶5,000; Sigma-Aldrich). A horseradish peroxidase-conjugated anti-rabbit or anti-mouse IgG was used as a secondary antibody, and signals were detected by chemiluminescence. Densitometric analyses were carried out using Multi Gauge software version 3.1 of the imaging system LAS-4000 (Fujifilm, Tokyo, Japan).

### Statistical analysis

The two-channel miRNA microarray data were background-corrected using the *normexp* [29] algorithm and then normalized using a global *loess* procedure [30]. miRNAs with a signal lower than the background in most samples were discarded. A linear model was fitted for each miRNA by fitting miRNA expression levels on two factors of interest – placental region and labor status. Moderated t-tests [31] were used to assess the significance of the effects, and correction for multiple testing was implemented. Significance was inferred by controlling the false discovery rate at 5%. All procedures were applied using the *limma* package of *Bioconductor* [32]. To compare the results of other experiments, Student's *t*-test or the Mann-Whitney U test for independent variables and the paired *t*-test or Wilcoxon signed rank tests for related variables were performed as appropriate, using the SPSS version 15.0 (SPSS Inc, Chicago, IL).

## Results

### MicroRNA expression profiling of the amnion

To determine region-specificity of the miRNA profile in the context of labor, expression of 875 human miRNAs was screened by microarray analysis of the placental amnion and the reflected amnion obtained from TNL and TIL cases. The complete microRNA microarray dataset is available in Gene Expression Omnibus (GEO; http://www.ncbi.nlm.nih.gov/geo/query/acc.cgi?token=xtebjacumgweulo&acc=GSE27441). A linear model-based analysis of microarray data showed differential expression of 32 miRNAs between the placental amnion and the reflected amnion in TIL cases (Table 2). Of note, 31 (97%) out of 32 differentially expressed miRNAs were down-regulated in the latter. Significant differential expression of miRNAs was not found in other comparisons: between the placental amnion and the reflected amnion in TNL cases, between the TNL placental amnion and the TIL placental amnion, and between the TNL reflected amnion and the TIL reflected amnion. Of note, the comparison between TNL reflected amnion and TIL reflected amnion demonstrated that the differences in the expression of 23 miRNAs had a raw p value less than 0.05 although they were not significant after adjustment for multiple comparisons. These 23 miRNAs are miR-517a, miR-519a, miR-521, miR-515-5p, miRPlus-E1170, miRPlus-E1112, miR-515-3p, miRPlus-E1108, miR-520c-3p, miR-518f*, miR-519b-3p, miR-512-3p, miR-517b, miR-143, miR-1323, miR-518f, miR-335, miR-145, miR-934, miR-99a, miRPlus-F1195, miR-1283, and miR-520d-5p. All of these miRNAs except miRPlus-E1112 were down-regulated in TIL cases.

**Table 2:** Differentially expressed miRNAs between placental amnion (PA) and reflected amnion (RA) with labor.

Term In Labor			
ID	*P* Value	Fold Change	Up-regulated in:
Has-miR-509-3p	0.011	2.55	PA
Has-miR-143	0.011	3.24	PA
Has-miR-431	0.011	3.47	PA
Has-miR-145	0.011	2.45	PA
Has-miR-409-3p	0.011	2.95	PA
hsa-miR-376b	0.013	2.38	PA
hsa-miR-654-3p	0.019	2.22	PA
hsa-miR-514	0.019	2.01	PA
hsa-miR-199a-5p	0.019	2.61	PA
hsa-miR-199a-3p/hsa-miR-199b-3p	0.020	2.71	PA
hsa-miR-379*	0.021	2.35	PA
hsa-miR-369-3p	0.022	2.10	PA
hsa-miR-154*	0.024	2.49	PA
hsa-miR-411*	0.024	2.50	PA
hsa-miR-487a	0.024	2.45	PA
hsa-miR-410	0.024	2.25	PA
hsa-miR-146b-5p	0.024	1.99	PA
hsa-miR-495	0.025	2.28	PA
hsa-miR-127-5p	0.031	2.69	PA
hsa-miR-377	0.031	1.94	PA
hsa-miR-146a	0.031	1.84	PA
hsa-miR-1308	0.032	2.00	PA
hsa-miR-199b-5p	0.032	2.05	PA
hsa-miRPlus-E1038	0.032	2.08	PA
hsa-miRPlus-E1170	0.038	1.79	PA
hsa-miR-376a	0.041	1.76	PA
hsa-miR-134	0.047	2.36	PA
hsa-miR-889	0.048	1.80	PA
hsa-miR-432	0.049	1.87	PA
hsa-miR-34a	0.049	2.14	PA
hsa-miR-1273	0.050	1.70	PA
hsa-miR-486-5p	0.044	1.76	RA

**Figure 1:**
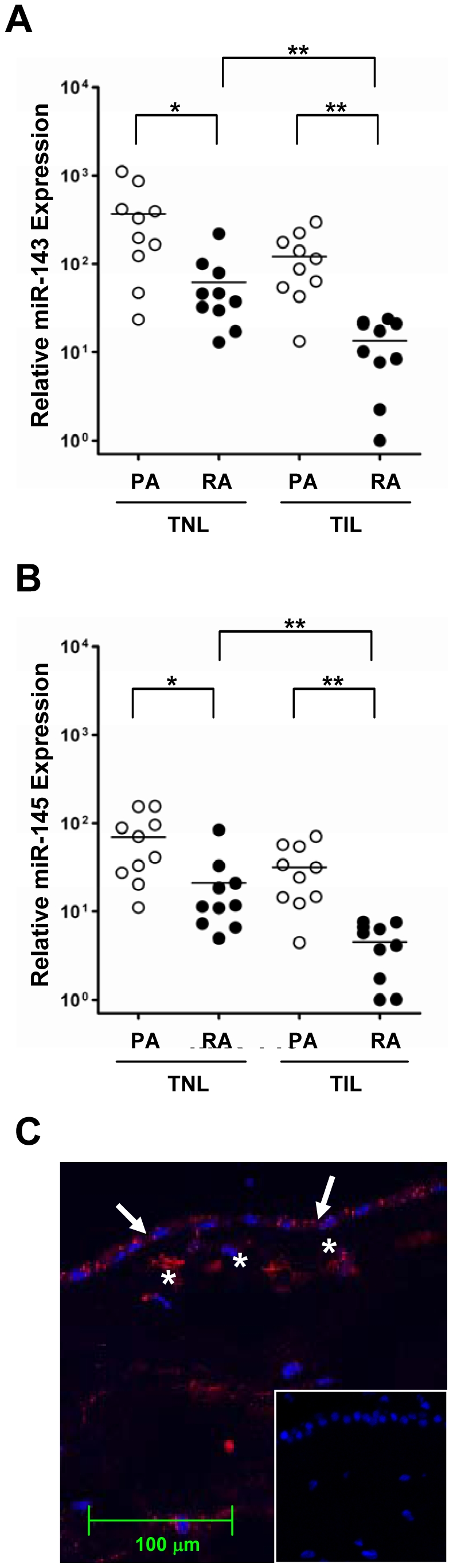
miR-143/miR-145 cluster expression in PA and RA. A, qRT-PCR analysis of miR-143 expression in PA and RA obtained from women at term not in labor (TNL; n = 10) and in labor (TIL; n = 10) to confirm microarray results. TNL cases are composed of 5 cases subjected to microarray analysis and 5 additional cases, while TIL cases are composed of 4 cases used in the microarray analysis and 6 additional cases because of RNA availability. miR-143 expression is significantly higher in the PA than in the RA in both groups, and its expression in the RA is significantly higher in TIL cases than in TNL cases. *, *p*<0.05 **, *p*<0.005. B, The differential expression patterns of miR-145 are basically identical to those of miR-143. C, *In situ* hybridization for miR-143 in the RA obtained from a TNL case. Hybridization signals are readily detected in the amnion epithelial cells (arrows) and mesenchymal cells (asterisks). Inset shows scrambled LNA *in situ* hybridization results used for negative control. Original magnification ×400. PA: placental amnion. RA: reflected amnion.

Because the miR-143/miR-145 cluster was also among the miRNAs whose differential expression had a raw p value of less than 0.05 between TNL reflected amnion and TIL reflected amnion as described above, expression of the cluster was subjected to further confirmation. When we confirmed their expressions by qRT-PCR, the miR-143/miR-145 expression was significantly lower in the reflected amnion of both TNL and TIL cases (*p*<0.05 for each); and its expression in the reflected amnion also significantly decreased with labor (*p*<0.05).The results were consistent and confirmed the microarray data. The miR-143 and miR-145 expressions were 7.7-fold and 5.7-fold higher in the placental amnion than in the reflected amnion of TIL cases, respectively (*p*<0.005 for each). The miR-143 and miR-145 expressions in the TNL reflected amnion were 3.2-fold and 2.4-fold higher than in the TIL reflected amnion, respectively (*p*<0.005 for each) (Figure 1A and 1B). Localization of miR-143 expression by *in situ* hybridization demonstrated readily detectable hybridization signals in both the amnion epithelial cells and the mesenchymal cells (Figure 1C).

### Prostaglandin-endoperoxidase synthase 2 as a putative target of miR-143

**Figure 2:**
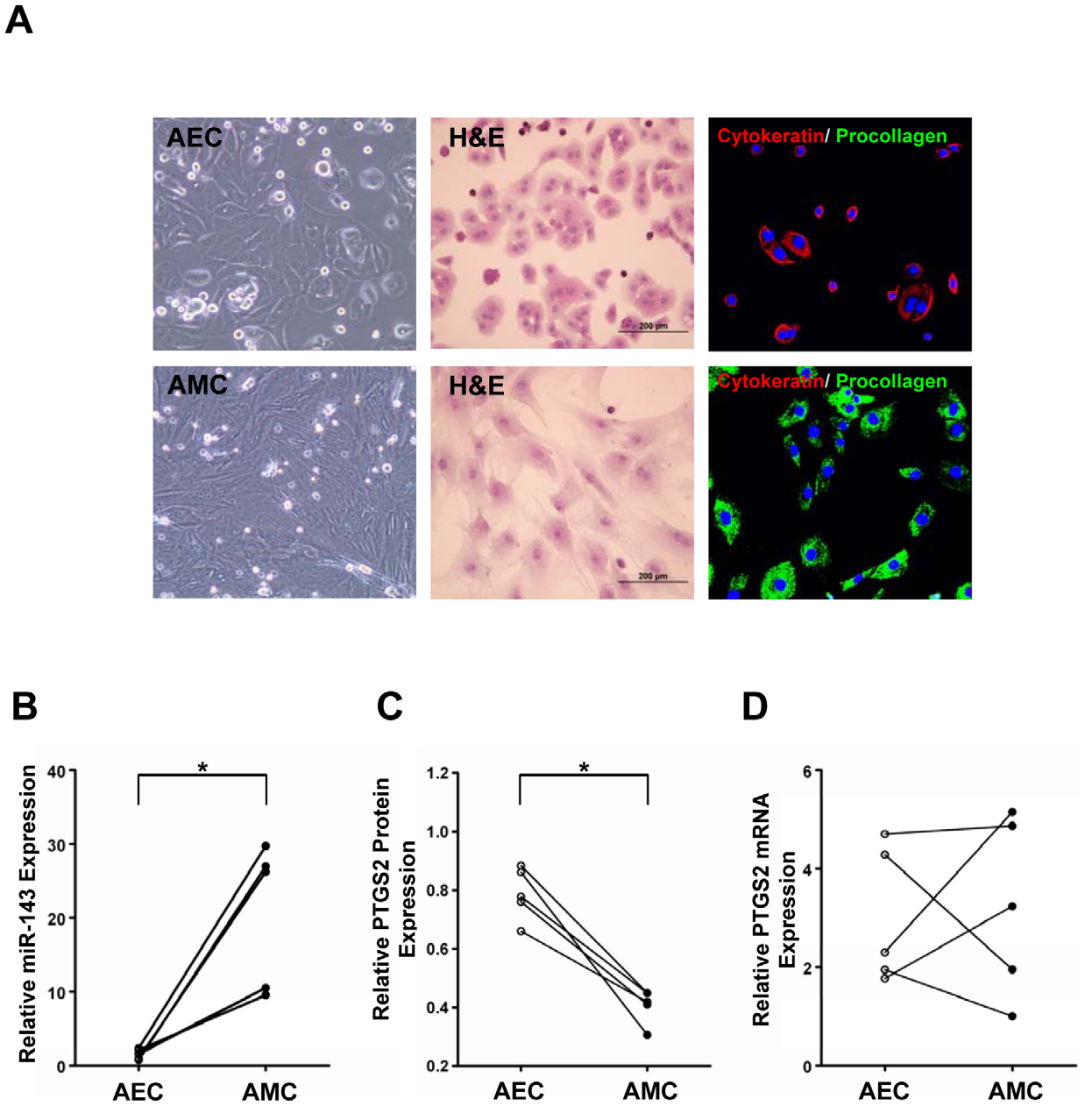
Characterization of isolated amnion epithelial cells (AECs) and amnion mesenchymal cells (AMCs). A, Morphological and immunophenotypic characteristics of AECs and AMCs on hematoxylin & eosin staining (H&E) and immunofluorescent staining of cytokeratin-7 (red), type I procollagen (green). AECs are positive for cytokeratin-7, while AMCs are positive for type I procollagen. B, qRT-PCR analysis of miR-143 expression which was normalized to 5S rRNA shows significantly higher expression in AMCs than AECs. C, Densitometric analysis of PTGS2 expression level was normalized to β-actin. PTGS2 protein is less abundant in AMCs than in AECs. D, PTGS2 mRNA expression is not different between AECs and AMCs. AECs and AMCs obtained from five women at term not in labor (TNL) were used for all experiments (B–D). *, *p*<0.05.

When we performed a computational search (http://pictar.mdc-berlin.de/) to determine a functionally relevant target in the context of labor, PTGS2, a key enzyme of labor at term, was among the putative targets of miR-143. As the amnion is composed of two different cell populations, and miRNA profile and function can markedly differ in various types of cells, we decided to study AECs and AMCs separately [33]. Immunofluorescence staining of isolated AECs and AMCs for cytokeratin-7 and type I procollagen demonstrated more than 95% purity for both populations of cells, AECs staining for cytokeratin-7 and AMCs for type I procollagen (Figure 2A).

Interestingly, qRT-PCR analysis showed a marked difference in miR-143 expression between AECs and AMCs. The miR-143 expression in AMCs was 15-fold greater (*p* = 0.028) than that of AECs (Figure 2B). On the other hand, densitometric analysis of PTGS2 protein expression of AMCs was 1.53-fold less (*p* = 0.043) than that of AECs (Figure 2C), while there was no difference in PTGS2 mRNA expression between AECs and AMCs (Figure 2D). These data suggested involvement of post-transcriptional regulation of PTGS2 expression.

### miR-143 binding to PTGS2 3′ UTR in amnion mesenchymal cells

**Figure 3:**
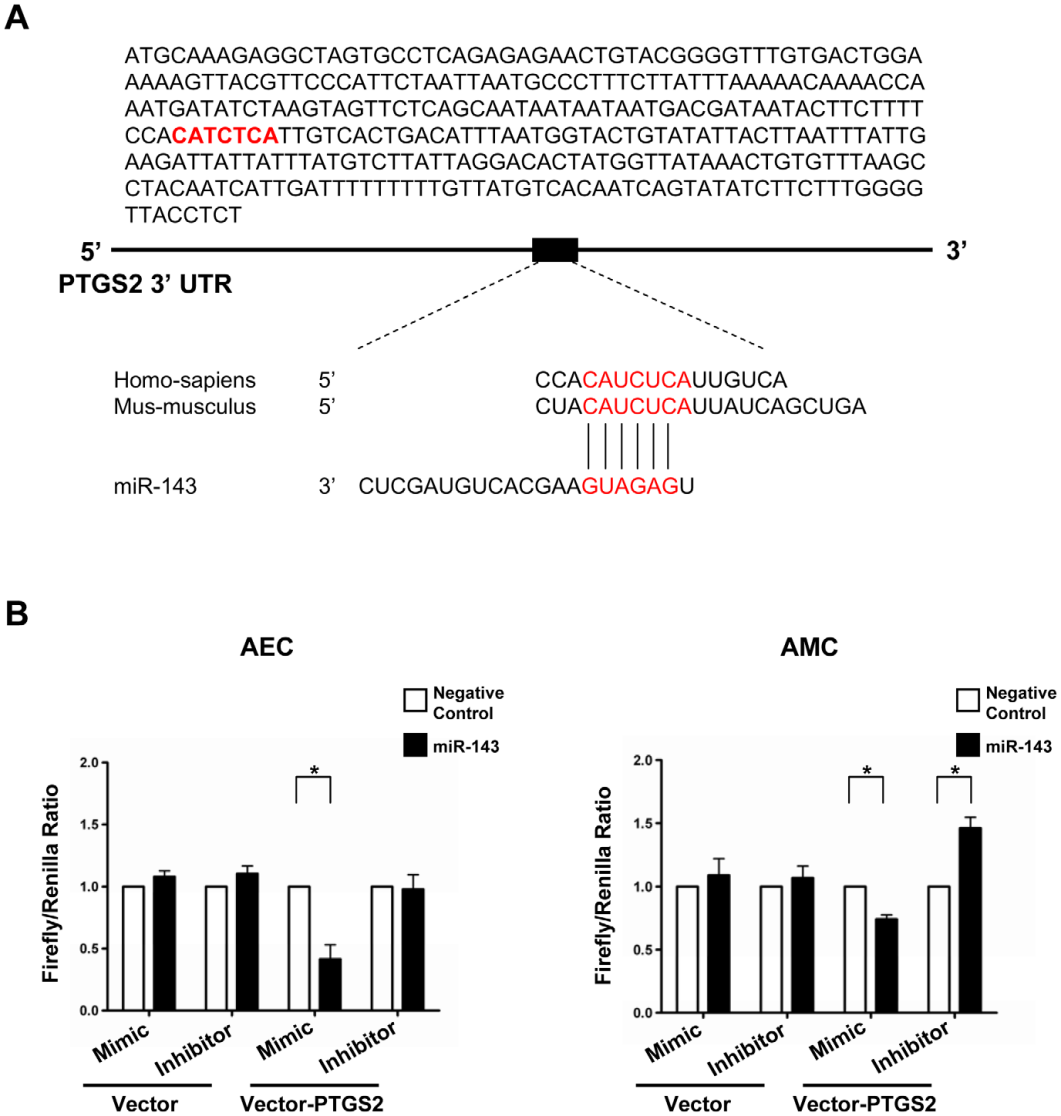
miR-143 binding to the 3′ UTR of PTGS2 mRNA. A, Putative miR-143-binding site in 333 bp of PTGS2 3′ UTR construct cloned into pMIR-REPORT™ for luciferase reporter assay. B, Luciferase reporter assay of PTGS2 mRNA 3′ UTR in AECs and AMCs. Luciferase activities from AECs and AMCs transfected with 200 ng (AEC) or 500 ng (AMC) of luciferase reporter plasmid containing PTGS2 3′ UTR (pMIR-REPORT_PTGS2), 10 ng of Renilla luciferase reporter pSV40-RL, and miRIDIAN miR-143 mimic (100 nM) or miR-143 hairpin inhibitor (50 nM) or equal amounts of negative controls were measured using Dual-Lucifrerase Reporter Assay System (Promega). pMIR-REPORT was used as a control. In AECs, there is a 58.5% of decrease in luciferase activity following transfection with miR-143 mimic but transfection of miR-143 hairpin inhibitor does not alter luciferase activity. In AMCs, miR-143 mimic transfection decreased luciferase activity by 25.9% compared to the control, while inhibition of miR-143 increased luciferase activity by 46.2% compared to control. Renilla luciferase activity was used for normalization of firefly luciferase activity (n = 5). The graphs show means and SE. *, *p*<0.05.

To confirm miR-143 binding to 3′ UTR of PTGS2 mRNA, a transient transfection experiment was carried out using a luciferase reporter plasmid with PTGS2 3′ UTR containing the putative miR-143 binding site (Figure 3A). Results of the luciferase assay were different between AECs and AMCs. In AECs, there was a decrease in luciferase activity by 58.5% (*p* = 0.008) following transfection with a miR-143 mimic (Figure 3B), but there were no significant changes in luciferase activity following transfection with a miR-143 hairpin inhibitor. Findings were most likely related to the low basal endogenous level of miR-143 expression in AECs. In AMCs, however, effects of both miR-143 mimic and hairpin inhibitor were readily detected (Figure 3B). While miR-143 mimic transfection decreased luciferase activity by 25.9% compared to the control (*p* = 0.008), inhibition of miR-143 resulted in a 46.2% increase (*p* = 0.008) in luciferase activity compared to the vector control (Figure 3C).

### miR-143 regulation of PTGS2 by translational repression

To determine whether post-transcriptional regulation of PTGS2 by miR-143 occurs via translational repression or mRNA degradation mechanisms [18], [34], AECs and AMCs were transfected with either miR-143 mimic or hairpin inhibitor, and the changes in miR-143 and PTGS2 expression following transfection were determined by qRT-PCR and immunoblotting, respectively. When AECs and AMCs were transfected with Cy3-labeled Pre-miRTM negative control #1, the efficiency of transfection was well over 80% in both cell types.

**Figure 4:**
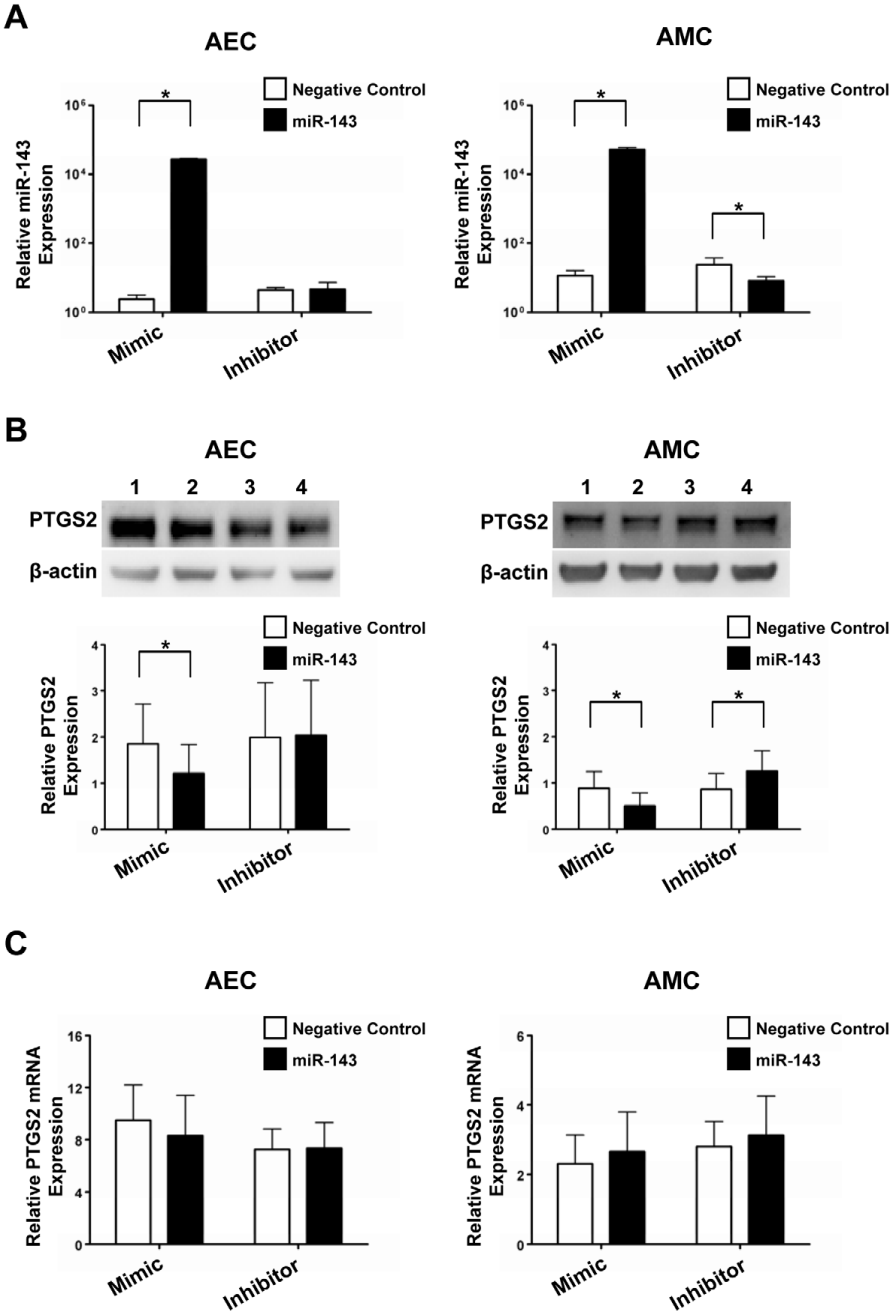
Effects of miR-143 on PTGS2 expression in AECs and AMCs. A, qRT-PCR analysis shows that 50 nM of miR-143 mimic transfection significantly increases miR-143 expression, while transfection with 200 nM of miR-143 inhibitor has no effect in AECs. In AMCs, miR-143 mimic (50 nM) and hairpin inhibitor (200 nM) transfections significantly increased and decreased the expression of miR-143, respectively. miR-143 and miR-145 expression levels were normalized with 5S rRNA expression. B, Immunoblot analysis of PTGS2 expression in AECs and AMCs transfected with mimic negative control (lane 1), mimic-miR-143 (lane 2), hairpin inhibitor negative control (lane 3), and inhibitor-miR-143 (lane 4). In AECs, PTGS2 protein expression was 1.5-fold decreased in transfection with miR-143 mimic (p = 0.014), but no changes in transfection with miR-143 inhibitor. In AMC, miR-143 mimic and hairpin inhibitor transfections 1.8-fold increased and 1.3-fold decreased the expression of PTGS2 protein (p = 0.014 for each), respectively. PTGS2 protein level was normalized to that of β-actin. C, qRT-PCR analysis of PTGS2 mRNA in transfected AEC and AMC shows there are no significant differences. The PTGS2 mRNA expression was normalized on the content of RPLPO. n = 4 for each experiments; the graphs show means and SE. *, *p*<0.05.

In AECs, miR-143 mimic transfection significantly increased (*p* = 0.014) miR-143 expression while decreasing PTGS2 expression. However, effects of miR-143 inhibitor transfection were minimal on miR-143 and PTGS2 expressions, which were consistent with the luciferase reporter assay data (Figure 4A and 4B). In AMCs, miR-143 mimic and hairpin inhibitor transfections significantly increased and decreased (*p* = 0.014 for both) the expression of miR-143, respectively (Figure 4A). Consequently, PTGS2 protein expression was decreased upon miR-143 mimic transfection, and inhibition of miR-143 increased PTGS2 expression (*p* = 0.014; Figure 4B). PTGS2 mRNA expression levels, however, in both AECs and AMCs (Figure 4C) were not significantly changed by miR-143 transfection. These results suggested that miR-143 regulation of PTGS2 expression occurs largely by translational inhibition than by PTGS2 mRNA degradation in the amnion, particularly in AMCs.

## Discussion

The role of the amnion in human parturition is critical, and all findings in the present study indicate that post-transcriptional regulation of amniotic gene expression by miRNAs is an integral component of human parturition. Primary findings of interest include: (1) the placental amnion and the reflected amnion of TIL cases also have distinct miRNA expression patterns as is the case with transcriptome; (2) the miR-143/miR-145 cluster is among the down-regulated miRNAs in the reflected amnion with labor; (3) miR-143/miR-145 expression is significantly higher in AMCs than in AECs; (4) miR-143 targets PTGS2 3′ UTR in amnion cells; and (5) miR-143 regulation of PTGS2 occurs largely by translational repression.

We have previously analyzed miRNA expression patterns in the chorioamniotic membranes including the amnion obtained from women at term not in labor, term in labor, and preterm labor with intact membranes, and found differential expression of miRNAs between term and preterm cases [15], [27]. Findings suggested that global down-regulation of miRNA in the chorioamniotic membranes precedes parturition at term. This study reports comparative microRNAome of the placental amnion and the reflected amnion for the first time, which revealed global down-regulation of differentially expressed miRNA in the reflected amnion compared to the placental amnion in the presence of labor at term. Therefore, human labor seems to be linked to de-repression of post-transcriptional inhibition of gene expressions by miRNA in the reflected amnion. This down-regulation of miRNA expression in the reflected amnion with labor could be attributed to the decreased expression of miRNA processing enzyme Dicer because decreased expression of Dicer is a feature of labor at term both in the amnion and the chorion [35].

Among miRNAs down-regulated with labor in the reflected amnion, we found the presence of miR-143 and miR-145 as well as their relative abundance in AMCs intriguing and relevant. The miR-143/miR-145 cluster has been widely studied in vascular smooth-muscle cells [36]. Cordes et al have demonstrated that the miR-143/miR-145 cluster is abundant in the developing heart and in their localization in smooth muscle cells and neural crest stem cell-derived vascular smooth muscle cells [37]. This cluster was the transcriptional target of the serum response factor, myocardin and NK2 transcription factor related, locus 5 (Nkx2-5), especially miR-145 could induce smooth muscle differentiation of neural crest stem cells. Conversely, this cluster targeted key cellular machinery, such as myocardin, Klf-4, and Elk-1, for smooth muscle differentiation. In miR-143/miR-145 knockout mice, vascular neointima formation after injury is blocked due to perturbations in actin stress fiber formation [38]. AMCs have a myofibroblast phenotype expressing smooth muscle actin, and acquire macrophage immunophenotype on meconium exposure or during inflammation [7]. Higher expression of miR-143/miR-145 in AMCs than in AECs, therefore, could be explained by intrinsic phenotype of the cells and is biologically quite relevant. In this context, it is also possible that the difference in miR-143/miR-145 expression between placental amnion and reflected amnion can be related to a potential difference in the ratio of amnion epithelial and mesenchymal cells between placental amnion and reflected amnion.

PTGS2 is a key enzyme in the synthesis of prostaglandins. There is good correlation between PTGS2 and PGE_2_ expression levels in the amnion [16]. Regulation of PTGS2 is a complex process involving both transcriptional and post-transcriptional events [39]. It has been shown that a complex of cytosolic proteins binds to an AU-rich sequence element in PTGS2 mRNA 3′ UTR and inhibits PTGS2 synthesis [40]. Chakrabarty et al recently demonstrated the functional significance of uterine expression of mmu-miR-101a and mmu-miR-199a and their regulation of PTGS2 in a murine embryo implantation model [41]. Findings in the present study indicate that the changes in miR-143 regulation of PTGS2 plays a role in labor at term in addition to the increase in transcription of PTGS2 mRNA. This miR-143 regulation is cell-type specific restricted to AMCs. The miRNAs which can potentially target PTGS2 are miR-199a*, miR-26a, miR-26b, miR-144, and miR-101, and it is noteworthy that miR-199a-5p and miR-199a-3p are also among the significantly down-regulated miRNAs in the reflected amnion of term in labor cases compared to the placental amnion.

The biological importance of other differentially expressed miRNAs in the present study needs further investigation. For example, miR-146a is one miRNA whose expression was lower in the reflected amnion, and it was shown to increase PGE_2_ expression in lung fibroblasts. miR-146a caused degradation of PTGS2 mRNA in lung fibroblasts, and its decrease increased the half-life of PTGS2 mRNA [42]. It has also been shown that miR-146a and Kruppel-like factor 4 constitute a feedback loop and that miR-146a promotes proliferation of vascular smooth muscle cells [43]. This miRNA is also a key molecule of innate immune response because it represses translation of interleukin 1 receptor associated kinase 1 (IRAK1), a critical mediator of Toll-like receptor signaling [44], [45].

In summary, all the findings herein clearly indicate that post-transcriptional regulation of gene expression by miRNA is important in human parturition, and this particularly seems to be the case in the reflected amnion. Furthermore, expression patterns of the miR-143/miR-145 cluster strongly suggest that miRNA-mediated regulation of gene expression in the amnion is a cell type-specific event. We report region-specific amniotic microRNAome (placental amnion vs reflected amnion) and miR-143 regulation of PTGS2 in amnion mesenchymal cells for the first time. This study reveals that there is a novel association linking a cellular differentiation program (AECs vs AMCs) and initiation of physiologic human labor.
